# The Role of Thymoquinone in Inflammatory Response in Chronic Diseases

**DOI:** 10.3390/ijms231810246

**Published:** 2022-09-06

**Authors:** Yan Liu, Lei Huang, Mi-Yeon Kim, Jae Youl Cho

**Affiliations:** 1 Department of Integrative Biotechnology, Sungkyunkwan University, Suwon 16419, Korea; 2 Department of Biocosmetics, Sungkyunkwan University, Suwon 16419, Korea

**Keywords:** thymoquinone, immunotherapeutic, inflammation, signaling pathways, chronic disease

## Abstract

Anti-inflammatory therapies have been shown to be effective in the prevention of various cardiovascular diseases, tumors, and cancer complications. Thymoquinone (TQ), the main active constituent of *Nigella sativa*, has shown promising therapeutic properties in many in vivo and in vitro models. However, TQ has poor bioavailability and is hydrophobic, prohibiting clinical trials with TQ alone. Studies have explored the combination of TQ with biological nanomaterials to improve its bioavailability. The TQ nanoparticle formulation shows better bioavailability than free TQ, and these formulations are ready for clinical trials to determine their potential as therapeutic agents. In this paper, we review current knowledge about the interaction between TQ and the inflammatory response and summarize the research prospects in Korea and abroad. We discuss the different biological activities of TQ and various combination therapies of TQ and nanomaterials in clinical trials.

## 1. Introduction

In recent years, an increasing number of chronic diseases, including cancers, autoimmune diseases, cardiovascular diseases, various infectious diseases, and neurodegenerative diseases, have received a great deal of attention from researchers working with chronic inflammation [1,2]. To relieve or prevent those diseases, relatively non-toxic natural drugs derived from plants and herbs have attracted attention for developing anti-inflammatory drugs and remedies. Plants with effective pharmacological components including saponins, alkaloids, and quinones have been extensively studied. Quinones have received research attention as the main pharmacological component of these [3].

**Figure 1:**
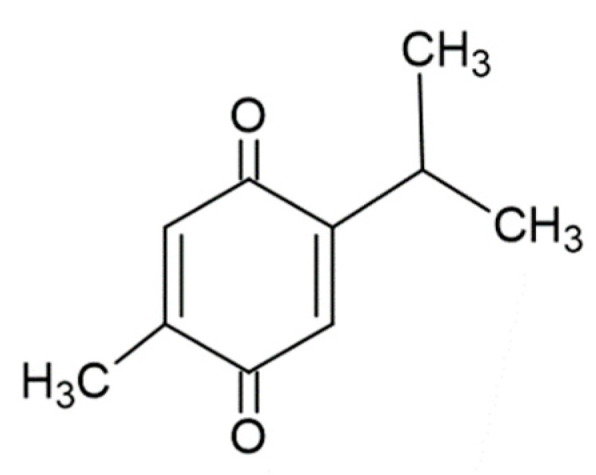
Chemical structure of thymoquinone (TQ).

The Ranunculaceae family of plants contains a class of organic compounds known as *p*-benzoquinones, one of the quinone compounds [4]. *Nigella sativa* (black cumin), a member of this plant family, is widely grown around South and Southeast Asia. The triangular seeds of this plant and their essential oils are the main source of thymoquinone (TQ: 2-isopropyl-5-methylbenzo-1, 4-quinone, MW = 164.2, Figure 1) [5].

TQ was first isolated in 1960 as a yellow precipitate slightly soluble in water and soluble in organic solvents such as ethanol, dimethyl sulfoxide, and dimethyl formamide. The pharmacological properties of TQ include anti-inflammatory, anti-tumor, immunostimulant, antioxidant, hepato-protective, and cardio-protective properties [6].

TQ has been extensively studied for its numerous biological activities in preclinical studies. Its potent antioxidant activity suggests a beneficial role in the control of many inflammatory diseases [7]. To date, only two phase 1 clinical studies have been conducted on TQ due to its low bioavailability. Nanotechnology can be used to overcome this problem through the development of TQ nanoparticles with better bioavailability than free TQ. For example, while curcumin and paclitaxel have bioavailability issues, nanocarrier formulation of these compounds has enabled their testing and ultimate FDA approval.

A wide variety of diseases is associated with the inflammatory response [8,9]. Inflammation has generally been described as either infectious or non-infectious. Signs of acute inflammation include redness, swelling, heat, pain, functional mask, and other changes; these signs are often accompanied by fever, leukocytosis, and other systemic reactions [10,11,12]. The emergence of these signs is a reflection of a struggle between the body and inflammatory factors. This struggle continues through the inflammatory process. Inflammatory factors cause cellular damage, leading to tissue necrosis; however, these factors also increase the body’s disease resistance function. In general, the disease-resistance function of inflammation outweighs its destructive properties. Inflammatory congestion can increase oxygen and nutrient supply to tissue surfaces, increase surface tissue metabolism, and increase tissue pathogen resistance force [13,14,15]. Exudates dilute toxins, and antibodies contained in the exudates defend against bacteria and neutralize toxins. The extravasated fibrin element of inflammation condenses into a false membrane and shelters against pathogens. Neutrophils and macrophages in the exudate phagocytize carriers and can engulf necrotic cell debris [10,16]. Plasma cells and lymphocytes produce antibodies to neutralize toxins, and tissue hyperplastic responses control the defects caused by inflammation [17].

**Figure 2:**
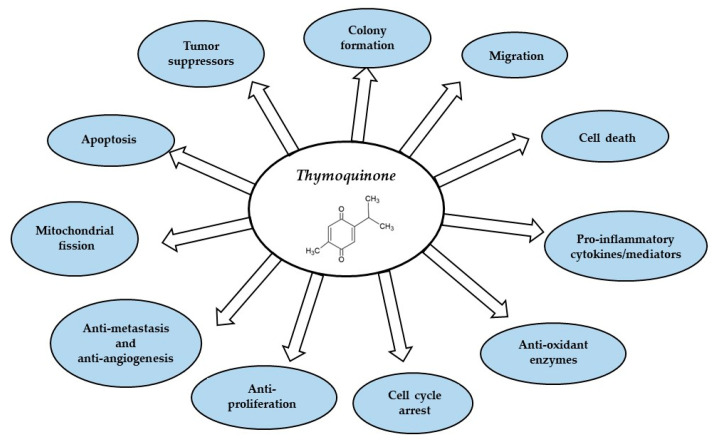
Thymoquinone targets different sites in inflammatory diseases and cancer.

In this review, we discuss the functional properties of TQ and its variety of pharmacological efficacies (Figure 2). We then comprehensively discuss the anti-inflammatory effect of TQ in different diseases and the role of TQ in vitro and in vivo. Our intent is to assist in providing a clear direction for future TQ research.

## 2. TQ Effect on Inflammation

Inflammatory responses are present in many diseases. These responses are a common pathological process that starts in the tissues and organs of the body. The anti-inflammatory effects of TQ involve multiple complex signaling pathways as well as molecular mechanisms, which mainly involve anti-inflammatory and antioxidant activity from the inhibition of oxidative stress [18].

Previous studies suggest that oxidative stress is one of the major causes of experimental autoimmune encephalomyelitis (EAE) progression [19]. In 2009, a study proposed the role of TQ in EAE. The authors used a C57BL/6J mouse model to simulate human multiple sclerosis (MS) and found that TQ was 90% preventive and 50% therapeutic, a large role in the fight against oxidative stress [20]. Amelioration of induced EAE symptoms by TQ administration has been shown in Lewis rats. The results demonstrated that 63% of model animals showed significantly improved clinical symptoms of EAE after TQ treatment and subsequent glutathione (GSH) 12–17 days later. Similarly, GSH prevented ROS-mediated oxidative stress damage, demonstrating that TQ can improve EAE and show a good therapeutic effect [21]. There is experimental evidence for the ability of TQ to increase the expression level of GSH. In one study using 120 one-day-old chickens, TQ was found to exhibit antioxidant properties by increasing the levels of GSH and glutathione-S-transferase enzyme alpha-3 (GSTA3) [22]. These results illustrate the effectiveness of TQ in preventing and treating EAE.

The anti-inflammatory effects of TQ are mainly exerted through cyclooxygenase-2 (COX-2). COX-1 and COX-2 are isoenzymes of COX, and COX-2 participates in the inflammatory response of peripheral blood inflammation [23]. COX-2 is closely related to the expression of growth factors and inflammatory factors in cells [24,25]. A study in streptozotocin-induced diabetic rats showed that TQ inhibits the expression level of lipid peroxidation malondialdehyde in the pancreas and reduces the superoxide dismutase antioxidant enzyme in tissues by increasing the expression level of COX-2 [26]. Similar results were observed in in vitro (HT-29 cells) and in vivo (mice) models. In vivo, TQ significantly reduced the disease activity index (DAI) and myeloperoxidase (MPO) activity, protecting the internal microenvironment of the colon. In vitro experiments demonstrated that TQ mediates COX-2 expression and exerts anti-inflammatory function by inhibiting MAPK and NF-κB signaling [27]. In 2013, the relationship between TQ and COX-2 was verified in the skin of female HR-1 hairless mice. The study results showed that TQ reduced NF-κB signaling gene expression while alleviating the increase of COX-2 in skin cells induced by 12-O-tetradecanoylphorbol-13-acetate (TPA) to activate the expression of skin cytoprotective proteins [28]. TQ also showed excellent antioxidant and antiarthritic activity in Wistar rats and reduced the expression of inflammatory factors such as IL-1β, TNF-α, IFN-γ, and IL-6 [29]. These findings indicate the important role of TQ in the anti-inflammatory process, and the signaling pathways involved have been explored. Despite these findings, the clinical application of TQ remains limited, so the clinical value of TQ requires further study.

In the following sections, we will focus on the anti-inflammatory effects of TQ in atherosclerosis (AS), myocardial infarction (MI), breast cancer, colorectal cancer (CRC), lung cancer, hepatocellular carcinoma (HCC), and neurodegenerative disorders.

## 3. Anti-Inflammatory Effects of TQ in Cardiovascular Disease (CVDs)

Cardiovascular disease (CVD) refers to cardiac and peripheral vascular diseases that generally result in ischemic or hemorrhagic diseases in the heart, brain, and systemic tissues caused by hyperlipidemia, blood alterations, atherosclerosis, and hypertension [30]. Cardiovascular disease is common and seriously threatens human health, especially in individuals older than 50 years. Cardiovascular disease is associated with high morbidity, disability, and mortality [31]. Even with the most advanced and complete treatments, more than 50% of cerebrovascular accident survivors have long-lasting dysfunction. The global population of individuals with cardiovascular and cerebrovascular diseases is as high as 15 million every year, with high mortality from a variety of causes. Cardiovascular disease is a multifactorial disease with complex etiology. The pathogenesis of CVD includes the deposition of lipids in the arterial vascular wall. These deposits form plaques that enlarge and thicken, narrowing the vascular diameter and decreasing blood flow and vascular elasticity. TQ may exhibit substantial effects in the control of inflammation in CVD (Table 1).

### 3.1. Atherosclerosis (AS)

Atherosclerosis is a widespread and progressive chronic arterial disease that is a leading cause of mortality and morbidity worldwide [8]. Some findings from APOE−/− mice suggest that TQ may be a therapeutic agent against cardiac injury caused by hypercholesterolemia and may alleviate atherosclerosis and other diseases [51]. The dysfunction of endothelial cells leads to oxidative stress and other reactions that precipitate the occurrence of CVDs such as atherosclerosis. Idris-Khodja et al. explored whether TQ could improve endothelial function in middle-aged rats. The research group compared young rats with middle-aged rats treated with TQ and found that TQ decreased angiotensin II expression, inhibited oxidative stress, and promoted angiotensin normalization [32]. In 2010, researchers found that TQ controlled the development of atherosclerosis in experimental rabbits. The results showed that TQ reduced atherosclerosis risk through an antioxidant pathway [33], although the exact mechanism remained unclear. In addition, TQ has demonstrated some biotoxicity, indicating the need for a more accurate mechanistic understanding of atherosclerosis.

### 3.2. Myocardial Infarction (MI)

Acute MI results in myocardial necrosis caused by acute, persistent ischemia and hypoxia due to coronary artery dysfunction. MI may be related to mitochondrial dysfunction, which can increase ROS and excess nitric oxide in the body [52]. In 2018, the possibility that TQ may inhibit MI was proposed by researchers. Cardiac ischemia/reperfusion (I/R) was performed in control and TQ-pretreated Wister rats, and hemodynamic parameters, MI area, and other MI indicators including the apoptotic level of cardiomyocytes were measured. TQ pretreatment significantly improved heart function, reduced infarct size and cardiac lactate dehydrogenase (LDH) and creatine kinase-MB (CK-MB) levels, and inhibited internal oxidative stress and apoptosis. The authors proposed that TQ can protect against and inhibit MI mainly through the activation of autophagy to produce antioxidant and anti-apoptotic effects [34]. In addition, the authors used a rat model to assess the levels of myocardial oxidative stress, inflammation, apoptosis, and autophagy in rats pretreated with TQ and ISO. The results showed that TQ pretreatment produced a dose-dependent reduction in the MI area and significantly reduced the elevation of serum cardiac markers caused by ISO. TQ also acted as an autophagy enhancer in repairing cardiomyocyte injury and dysfunction [53]. Using the same approach, Medhet et al. confirmed that the antiapoptotic activity and inhibitory modulation of MMP-9 expression by TQ contribute to its protective effect in MI [35]. In summary, TQ can improve cardiac function and relieve MI through the inhibition of oxidative stress caused by MI and the increased activity of myocardial-related enzymes. The main pathway of TQ in inducing these effects is through antioxidants to inhibit cardiomyocyte apoptosis.

## 4. Anti-Inflammatory Effects of TQ in Tumors

Cancer diseases have a high mortality rate globally. The current main methods for cancer treatment include chemotherapy, radiotherapy, and surgical resection. However, these treatment options are frequently ineffective and have significant negative sequela. The development of post-treatment secondary tumors is also a concern. Anti-tumor vaccines and anti-tumor drug development are now under exploration [17,54]. TQ is a potential anti-cancer drug with prominent anti-tumor and antioxidant effects, and its anticancer properties involve multiple cellular pathways.

### 4.1. Breast Cancer

Breast cancer is the most common malignancy in females worldwide [55]. TQ exhibits marked therapeutic effects on human breast cancer cells [56]. Recently, studies have shown the combined effects of natural bioactive compounds including curcumin and TQ on MCF7 and MDA-MB-231 breast cancer cell lines [36]. Curcumin and TQ induced apoptosis and cell cycle arrest and reduced cancer cell proliferation, colony formation, and migration in breast cancer cells [36]. Subsequently, Sjs et al. developed a nanomedicine with TQ that induced DNA damage and apoptosis, inhibited cell proliferation, and prevented cell cycle progression [37].

Their work verified the excellent anticancer properties of TQs [57]. Murphy et al. has also proposed a targeted delivery method using phospholipid shells to deliver TQ to breast tumor lesion sites based on its natural hydrophobicity [56]. This novel formulation offers great potential for targeted delivery of hydrophobic chemotherapeutic agents to MDA-MB-231 breast cells as well as breast tumors [56]. These studies provide evidence that TQ may represent a novel strategy to alleviate and treat breast cancer.

### 4.2. Colorectal Cancer (CRC)

CRC is one of three major causes of death worldwide involving energy metabolism disorder in human cells [58]. One study proposed that TQ can effectively target and eliminate CRC cells, mainly by associating with cell cycle arrest and DNA damage in CRC cells [38,59]. In addition, a combination of TQ with ionizing radiation reduced DNA repair and stemness of CRC [38]. TQ is an important component in the regulation of metabolism in CRC. TQ may inhibit carcinogenesis and the development of CRC by regulating the glycolytic metabolic pathway and regulating the PI3/AKT axis. TQ was found to function as an antimetabolic drug in clinical studies [39].

### 4.3. Lung Cancer

As one of the cancers with a lower survival rate, lung cancer has been challenged with many different trials with new generations of chemical drugs and promising immunotherapeutic approaches with antibodies to lead to the successful treatment of this tumor [60,61]. Among them, drug intervention is also a hot spot in lung cancer treatment [62].

Khan et al. demonstrated that TQ-PSL pretreatment restored the relative weight of the lung, reduced the marker enzymes of cancer, and increased the activity of antioxidant enzymes in serum [63]. Histopathological analysis indicated that TQ-PSL protected against malignancy in the lung [63]. TQ may be a promising treatment for lung adenocarcinoma. TQ inhibited tumor cell proliferation by causing lung cancer cell apoptosis, significantly arresting cells in the S phase of the cell cycle, and significantly reducing the activity of TNF-α and NF-κB [40,64]. TQ has also been widely used in biomaterial treatments. Combining TQ nanoparticles with the TKI gefitinib may provide an effective platform for treating non-small cell lung cancer (NSCLC). Nanoparticles containing TQ showed better anti-lung cancer activity and induced therapeutic effects of epithelial-to-mesenchymal transition (EMT) in NSCLC by regulating the STAT3/PTEN/AKT/miR-21 axis [41]. These studies indicate an important role of TQ in lung cancer treatment.

### 4.4. Hepatocellular Carcinoma (HCC)

A recent study showed that the application of TQ with inhibitors at the initial stage of diethylnitrosamine (DEN)-induced HCC in rats resulted in the prevention of carcinoma progression. TQ pretreatment in rats prevented histopathology and most of the biochemical changes and improved the antioxidant status [42,43]. TQ also induces HCC cell death, activates the ERK and p38 signaling pathways, and exhibits excellent anti-HCC potential under suitable p-ERK inhibition conditions [42,43]. Zhang et al. proposed that TQ can cooperate with TRAIL to induce apoptosis in HCC through the mediation of DNA damage [65].

### 4.5. Other Cancers

TQ also has some effects on other cancers. For example, TQ significantly increased the level of ROS production in human pancreatic cancer cells and inhibited cancer cell proliferation and migration. These findings indicate that naturally existing quinones such as TQ may be effective anti-metastatic agents for pancreatic cancer [44]. TQ has also been shown to effectively inhibit the fusion of autophagosomes and lysosomes, leading to apoptosis in cancer cells. Moreover, TQ also initiated the miR-877-5p and PD-L1 signaling pathways, leading to inhibition of the migration and EMT of bladder cancer cells. In addition, TQ induces apoptosis by upregulating ROS levels [45].

## 5. Anti-Inflammatory Effects of TQ in Neurodegenerative Disorders

Neurodegenerative diseases are caused by the loss of neurons and their myelin sheaths, with deterioration and dysfunction occurring over time [66]. The main factor causing neurodegenerative diseases is oxidative damage of neural tissue caused by oxidative stress. The second factor is mitochondrial dysfunction caused by deletions or mutations of genes leading to apoptosis and neuronal damage [67]. The third main factor is a high concentration of glutamate that is toxic to neurons and leads to their senescence and death [67]. The fourth and most important cause of oxidative stress is an innate immune system inflammatory response that causes brain damage [68]. A recent study showed that TQ can act as a natural oxidant in the rat brain. The authors first used alumina to induce neurotoxicity and then observed the psychomotor conditions and oxidative inflammation levels of male albino rats through the action of TQ. The authors confirmed that TQ effectively alleviated rat neurotoxicity induced by alumina [69].

Neurodegenerative diseases include Alzheimer’s disease (AD), Parkinson’s disease (PD), Huntington’s disease (HD), amyotrophic lateral sclerosis (ALS), and different types of spinocerebellar ataxia (SCA), among others. Here, we discuss the actions of TQ in AD, PD, and HD.

### 5.1. Parkinson’s Disease (PD)

PD is a chronic disease of the central nervous system that affects the mobility of patients and mostly occurs in middle-aged and elderly people [70]. Ardah et al. constructed a mouse model of PD and confirmed that TQ reduced oxidative stress and neuro-inflammation induced by 1-methyl-4-phenyl 1,2,3,6 tetrahydropyridine (MPTP) [46]. The method was assessed by detecting the reduced activity of superoxide dismutase and catalase. Moreover, TQ significantly reduced the expression of COX-2 and inducible nitric oxide synthase (iNOS) [46]. According to the test that caused a decreased memory in animals, TQ was found to enhance the levels of amyloid beta 1-42 (Aβ 1-42) peptides injected into the hippocampus and improve the memory of rats. The administration of TQ significantly reduced the expression of Aβ, phosphorylated-tau, and BACE-1 proteins [47].

### 5.2. Alzheimer’s Disease (AD)

AD and the associated dementia have a high incidence and an insidious onset [71]. Clinically, AD is characterized by generalized dementia manifestations including memory impairment, aphasia, misuse, agnosia, visual-spatial skill impairment, executive dysfunction, and personality and behavioral changes [72,73]. BV-2 microglial cells were activated with lipopolysaccharide (LPS) and interferon-gamma (IFN-γ), and TQ (12.5 M, 24 h) was applied to treated cells [48]. The expression of neuroprotectin was significantly increased, while some associated inflammatory factors were significantly reduced. However, expression of the signaling target genes of the NF-κB pathway was also significantly reduced [48]. Therefore, TQ significantly inhibited neurotoxicity and neuroinflammation. The activation of the NF-κB pathway was reduced, protecting the nerves from damage [48,74]. Furthermore, TQ doses of 10, 20, and 40 mg/kg/day administered in AD model rats significantly reduced the expression of TLR2, TLR4, myeloid differential factor 88, tumor necrosis factor-alpha (TNF-α), interleukin-1beta (IL-1β), interferon-β, interferon regulatory factor 3 (IRF-3), and nuclear factor-κB (NF-κB) [49], as similar seen in LPS/IFN-γ-activated BV-2 microglia cells [48]. Therefore, these findings indicate that TQ can effectively alleviate pathological symptoms and neuronal damage in AD.

### 5.3. Huntington’s Disease (HD)

HD is a rare autosomal dominant disorder generally developing in middle age, with motor, cognitive, and psychiatric symptoms [75]. The onset is insidious, and the progression is slow. Robot-like movements with progressive cognition and mental dysfunction and eventual dementia are the main characteristics of the disease. The etiology is misexpression of polynucleotide repeats in the Huntington gene, which affects molecular pathways and ultimately leads to neural dysregulation and degeneration. Administration of 3-nitropropionic acid (3-NP) was found to induce HD dysfunction in male Wistar rats. TQ was used to treat these rats, and the expression of glial fiber acidic protein (GFAP) and proinflammatory cytokines and p-p65 NF-κB nuclear translocation was reduced [50].

## 6. Anti-Inflammatory Effect of TQ in Other Diseases

TQ also plays anti-inflammatory roles in a variety of diseases including sepsis, rheumatoid arthritis, obesity, and infectious diseases [76,77,78]. However, the underlying mechanisms remain unclear and require further study. A critical recent finding is that TQ has a promising effect on COVID-19 [79,80]. Because of the increasing prevalence of COVID-19, associated research has become popular.

In addition to the direct anti-COVID activity of TQ, many studies also showed that TQ can be effective in preventing cardiovascular complications caused by COVID-19 [30]. TQ is considered in CVD because it can activate endothelial cells by increasing the production of NO and endothelial cell-derived hyperpolarization factor (EDHF) in vivo and reducing vasoconstrictor factor (thromboxane A2) and oxidative stress in vascular smooth muscle cells. Therefore, these reports suggest TQ as a promising drug to treat various symptoms of COVID-19 and its cardiovascular complications.

## 7. Conclusions and Perspectives

**Figure 3:**
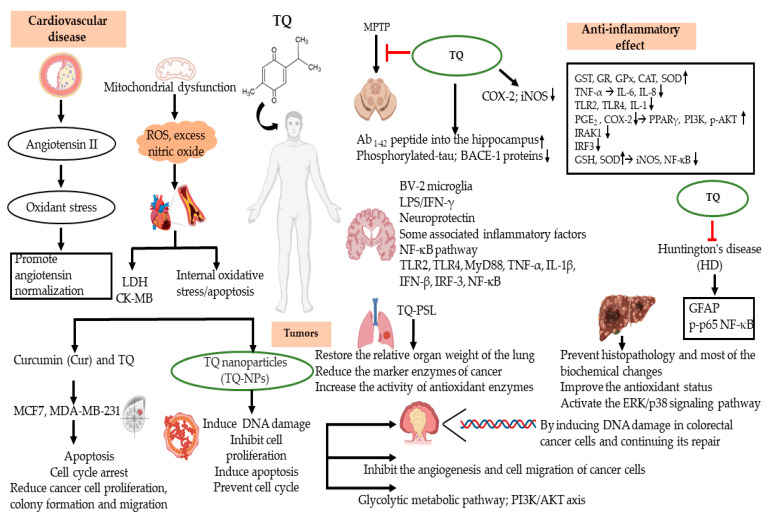
TQ displays curative activity against chronic diseases such as cancers, autoimmune diseases, cardiovascular diseases, and neurodegenerative diseases by suppression of inflammatory responses and oxidative stress.

This review describes in detail the anti-inflammatory and antioxidant mechanisms of TQ in different diseases. From these previous studies, we believe that TQ plays a powerful antioxidant role in the pathogenesis of disease. The properties of TQ have been demonstrated in the treatment of CVDs, cancers, and neurodegenerative diseases. Atherosclerosis, a CVD, is mainly caused by endothelial injury accompanied by many inflammatory reactions. These reactions result in the oxidation of low-density lipoprotein (LDL) by macrophages to form peroxide and superoxide ions, SMC proliferation, and plaque formation in the vascular wall [81]. TQ was found to be involved in this process and is mainly effective in reducing the level of LDL to prevent foam cell formation through an antioxidant function. Based on in vitro and in vivo studies, TQ may be a beneficial treatment for several inflammatory disorders, as summarized in Figure 2 and Figure 3.

Previous studies have confirmed that TQ is involved in the regulation of various molecular signaling pathways, mainly inflammatory signaling pathways. For example, the NF-κB signaling pathway is inhibited by TQ, while the Nrf2-ARE pathway is activated, and the expression of PI3K/protein kinase B is decreased. TQ can also alleviate oxidative stress response by reducing ROS production, mainly by inhibiting the expression of various pro-inflammatory factors, including IL-1β, IL-6, TNF-α, IFN-γ, and PGE_2_. Because of its anti-inflammatory properties, TQ is regarded to have protective effects against CVD, neuroinflammation, cancer, and heart-related diseases. How TQ suppresses inflammatory responses is not yet fully elucidated. Therefore, the molecular and cellular mechanisms of TQ-mediated anti-inflammatory activity should be further explored.

The bioavailability of TQ is very low. Although many studies have reported on TQ, few have explored the clinical application of TQ due to its low oral adsorption. To solve this problem, many researchers have employed nanotechnology to develop nanocarriers to improve the bioavailability of TQ, as with curcumin and paclitaxel. These approaches have shown that biological nanomaterials based on TQ can achieve more effective results in the prevention and treatment of diseases than TQ alone. More clinical trials using nanocarrier preparations of TQ should be conducted, keeping in mind that combination therapy can improve the efficacy of TQ. Because the biological characteristics of TQ mainly include anti-inflammatory and anti-oxidative properties, nanocarrier delivery of TQ may play a key role in the treatment of chronic diseases caused by sustained inflammation. In addition, a recent study demonstrated the killing activity of TQ against COVID-19 [82]. TQ may thus represent a new approach to developing a new class of anti-viral drugs. Therefore, the targeted delivery of TQ combined with biological nanomaterials will become a research hotspot to treat various kinds of human chronic diseases. Finally, it is noteworthy that only a few studies have adequate control (Table 1), indicating that additional experiments with appropriate controls should be prioritized in the future to provide more reliable information on TQ.

## Figures and Tables

**Table 1:** The pharmacological activity and mechanism of TQ in various diseases.

Disease Type	Disease	Mechanisms	Control Type	Test Type	References
Cardiovascular	Atherosclerosis (AS)	TQ decreases angiotensin II expression, inhibits oxidative stress, and promotes angiotensin normalization. TQ reduces lipid accumulation and enhances antioxidant capacity and renal function.	3% ethanol; EEP (75 mg/kg)	In vivo and in vitro	[32,33]
Cardiovascular Tumors	Myocardial Infarction (MI)	TQ improves heart function, reduces infarct size, reduces cardiac LDH and CK-MB, and inhibits internal oxidative stress and apoptosis. TQ antiapoptotic activity and inhibitory modulation of MMP-9 expression contribute to the TQ protective effect in MI.	Vehicle (100 μL/kg); Saline	In vivo and in vitro; In vivo	[34,35]
	Breast cancer	TQ induces apoptosis and cell cycle arrest; reduces cancer cell proliferation, colony formation, and migration; and demonstrates promising anticancer benefits in breast cancer. TQ induces DNA damage, inhibits cell proliferation, induces apoptosis, and prevents cell cycle progression.	DMSO	In vitro	[36,37]
Tumors Neurodegenerative disorders	Colorectal Cancer (CRC)	TQ inhibits the angiogenesis and cell migration of cancer cells. TQ inhibits the carcinogenesis and development of colorectal RC by regulating the glycolytic metabolic pathway and the PI3/AKT axis.	Methanol; 3-bromopyruvate	In vitro	[38,39]
	Lung cancer	TQ inhibits tumor cell proliferation by causing lung cancer cell apoptosis to significantly arrest the S phase cell cycle and significantly reduce the activity of TNF-a and NF-κB to achieve anticancer effects. TQ regulates the STAT3/PTEN/AKT/miR-21 axis.	PLGA nanoparticles; Normal saline (1 mL/kg/day)	In vitro; In vivo and in vitro	[40,41]
	Hepatocellular carcinoma (HCC)	TQ pretreatment in rats can prevent histopathology and most of the biochemical changes and improve the antioxidant status. TQ induces HCC cell death and activates the Erk and p38 signaling pathway. TQ exhibits excellent anti-HCC potential under suitable p-Erk inhibition conditions. TQ cooperates with TRAIL to induce apoptosis in cancer cells, mediated through DNA damage.	DMSO	In vivo and in vitro	[42,43]
	Pancreatic cancer	TQ significantly increases the level of ROS production in human pancreatic cancer cells and inhibits cancer cell proliferation and migration.	DMSO	In vivo	[44]
	Bladder cancer	TQ inhibits the fusion of autophagosomes and lysosomes, leading to apoptosis in cancer cells. TQ initiates the miR-877-5p and PD-L1 signaling pathways, inhibiting the migration and EMT of bladder cancer cells.	DMSO	In vivo and in vitro	[45]
	Alzheimer’s disease (AD)	TQ reduces activity of superoxide dismutase and catalase and the expression of COX-2 and iNOS. TQ enhances the Aβ1-42 peptides injected into the hippocampus and improves the memory ability of rats. TQ significantly reduced the expression of Aβ, phosphorylated-tau, and BACE-1 proteins.	0.9% NaCl saline solution	In vitro	[46,47]
Neurodegenerative disorders	Parkinson’s disease (PD)	TQ inhibits activation of the NF-κB pathway. TQ reduces the expression of TLR-2, TLR-4, MyD88, TNF-α, IL-1β, IFN-β, IRF-3, and NF-κB.	Normal saline and corn oil; 0.9% saline	In vivo	[48,49]
	Huntington’s disease (HD)	TQ attenuates the expression of GFAP, proinflammatory cytokines, and p-p65 NF-κB nuclear translocation.	0.9% saline	In vivo	[50]

## Data Availability

The data are contained within the article.

## Abbreviations

TQ	Thymoquinone, 2-isopropyl-5-methylbenzo-1,4-quinone
GST	Glutathione S-transferase
GR	Glutathione reductase
GPx	Glutathione peroxidase
CAT	Catalase
EAE	Experimental autoimmune encephalomyelitis
MS	Multiple sclerosis
GSH	Glutathione
GSTA3	Glutathione-S-transferase enzyme alpha-3
MPO	Myeloperoxidase
DAI	Disease activity index
TPA	12-O-tetradecanoylphorbol-13-acetate
AS	Atherosclerosis
MI	Myocardial Infarction
CRC	Colorectal Cancer
HCC	Hepatocellular carcinoma
AD	Alzheimer’s disease
PD	Parkinson’s disease
NO	Nitric oxide
CCL12	Chemokine C-C motif ligand 12
MCP-5	Monocyte chemotactic protein 5
GCSF	Granulocyte colony-stimulating factor
mPGES-1	Microsomal prostaglandin E synthase-1
COX-2	Cyclooxygenase-2
IL-1β	Interleukin 1 β
IL-6	Interleukin 6
IFN-γ	Interferon gamma
TNF-α	Tumor necrosis factor alpha
PGE_2_	Prostaglandin E2
LPS	Lipopolysaccharides
ARE	Antioxidant responsive element
NFE2L2/Nrf2	Nuclear factor (erythroid-derived 2) -like 2
PI3K	Phosphoinositide 3-kinase
PKB	Protein kinase B
MPTP	1-methyl-4-phenyl 1,2,3,6 tetrahydropyridine
iNOS	Nitric oxide synthase
Aβ1-42	The amyloid beta 1-42
